# Different Synthesis Protocols for Co_3_O_4_–CeO_2_ Catalysts—Part 1: Influence on the Morphology on the Nanoscale

**DOI:** 10.1002/chem.201403636

**Published:** 2014-11-10

**Authors:** Jingxia Yang, Liliana Lukashuk, Johanna Akbarzadeh, Michael Stöger-Pollach, Herwig Peterlik, Karin Föttinger, Günther Rupprechter, Ulrich Schubert

**Affiliations:** [a] Institute of Materials Chemistry, Vienna University of Technology 1060 Wien (Austria), Fax: (+43) 1-5880115399 E-mail: ulrich.schubert@tuwien.ac.at; [b] Faculty of Physics, University of Vienna 1090 Wien (Austria); [c] University Service Center for Transmission Electron Microscopy, Vienna University of Technology 1040 Wien (Austria)

**Keywords:** catalytic materials, cerium, cobalt, nanoparticles, sol–gel processes

## Abstract

Co_3_O_4_-modified CeO_2_ (Co/Ce 1:4) was prepared by a combination of sol–gel processing and solvothermal treatment. The distribution of Co was controlled by means of the synthesis protocol to yield three different morphologies, namely, Co_3_O_4_ nanoparticles located on the surface of CeO_2_ particles, coexistent Co_3_O_4_ and CeO_2_ nanoparticles, or Co oxide structures homogeneously distributed within CeO_2_. The effect of the different morphologies on the properties of Co_3_O_4_–CeO_2_ was investigated with regard to the crystallite phase(s), particle size, surface area, and catalytic activity for CO oxidation. The material with Co_3_O_4_ nanoparticles finely dispersed on the surface of CeO_2_ particles had the highest catalytic activity.

## Introduction

In most sol–gel procedures for mixed-metal oxides, the metal precursors are simply mixed. As the hydrolysis and condensation rates of the precursors can differ considerably, phase separation, however, can occur, which adversely affects the advantages of sol–gel processing. We have previously shown that a possibility to overcome these shortcomings is the use of single-source precursors in which the two metals are interlinked. To this end, precursors of the type (RO)_*n*_M-l-X-L′-M′ are required in which M(OR)_*n*_ is a metal alkoxide moiety, M′ the second metal (metal ion or another metal alkoxide moiety), L and L′ are the coordinating groups, and X is a chemically inert spacer. This synthetic route proved to be very efficient for silica-based mixed-metal oxides (i.e., M=Si), because a variety of alkoxysilanes (RO)_3_Si(CH_2_)_3_L′ with a coordinating group L′ are available.[1] Preparation of mixed oxides of any two metallic elements by this approach is less straightforward, because bifunctional organic compounds l-X-L′ with two different coordinating groups (L and L′) are required that must selectively react with just one metal component. As a proof of concept, we have demonstrated for M=Ti(O*i*Pr)_*x*_ and M′=Zn^2+^ that this approach results indeed in a more homogeneous distribution of both metals during sol–gel processing.[2]

In this article, we are comparing the outcome of this single-source precursor (SSP) approach with that of two more conventional sol–gel approaches for mixed-metal oxides. The chosen system, namely, Co_3_O_4_–CeO_2_ is an interesting CO, hydrocarbon, and diesel soot oxidation catalyst.[3] The high activity of Co_3_O_4_–CeO_2_ catalysts is attributed to synergetic interactions between cobalt oxide and ceria, which modify the Ce^3+^/Ce^4+^ and Co^2+^/Co^3+^ redox cycles and increase oxygen mobility as a result. This renders more oxygen available for the oxidation processes and improves the catalytic activity.[4] The dispersion of the oxides and a high specific surface area are critical for the catalytic performance of the material.[5]

We will show that the morphology of the Co_3_O_4_–CeO_2_ composites and their structure on the nanoscale is indeed influenced by variations of the preparation method, and we present preliminary data on catalytic CO oxidation reactions. In a follow-up paper (Part 2[6]) we will investigate in great detail how the catalytic properties are influenced by the different morphologies.

## Results and Discussion

In a previous work we reported that ceria with high surface area (up to 277 m^2^g^−1^) and small particle diameters (3–6 nm) can be prepared from acetaldoximate-modified cerium *tert-*butoxide (CeB) by a combination of sol–gel processing and solvothermal treatment. This work also showed that the precursor composition as well as the processing parameters had a strong influence on the properties of the materials. Modification of CeB with acetaldoxime (AO) [Eq. (1)] resulted in materials with superior properties, especially when combined with the nonionic surfactant Pluronic F127 as a pore-forming agent.[7] Substitution of metal alkoxides by bidentate ligands (such as oximate) not only moderated the reaction rates of metal alkoxides, but also resulted in additional porosity/surface area upon heat treatment of the gels.[7, 8]


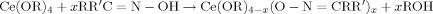
(1)

We therefore employed acetaldoximate-modified CeB and the surfactant F127, as well as the reaction and processing conditions optimized for ceria (solvothermal treatment in ethanol (STE) followed, optionally, by calcination in air (AC) for removal of organic groups) also for the synthesis of high surface-area Co_3_O_4_–CeO_2_. Cobalt acetate, Co(OAc)_2_, was taken as a Co source. We have previously shown that co-processing of metal alkoxides and metal acetates results in mixed-oxide structures.[9]

Three different variations of sol–gel processing were chosen, which differ by the way in which Co^2+^ ions are introduced into the gels. In route 1 (samples labeled **1**), a ceria gel was synthesized first. Co(OAc)_2_ was then added to the gel, and the mixture was solvothermally treated in ethanol (the solvothermally treated samples are labeled *x*-STE). In route 2 (samples labeled **2**), a mixture of CeB and Co(OAc)_2_ was subjected to sol–gel processing and then treated solvothermally. In route 3 (samples labeled **3**), CeB and Co^2+^ ions were interlinked by means of *p*-carboxybenzaldehyde oxime (POBC–H) to form a single-source precursor, which was then subjected to sol–gel processing and solvothermal treatment. To investigate the influence of the organic groups, part of the samples (**1**–**3**) was heat-treated in air at 500 °C after solvothermal treatment. These samples are labeled *x*-STE-AC.

The Co/Ce ratio was of 1:4 in all samples. The chemical ratio of the precursor components was the same, namely, CeB/Co(OAc)_2_/oximate groups/F127=0.8:0.2:1.6:0.005. Whereas AO was the only oximate group for routes 1 and 2, both POBC and AO were the oximate groups with a 1:3 ratio for route 3 (see the Experimental Section).

### Synthesis of the Ce-L-X-L′-Co single-source precursor

**Figure 1:**
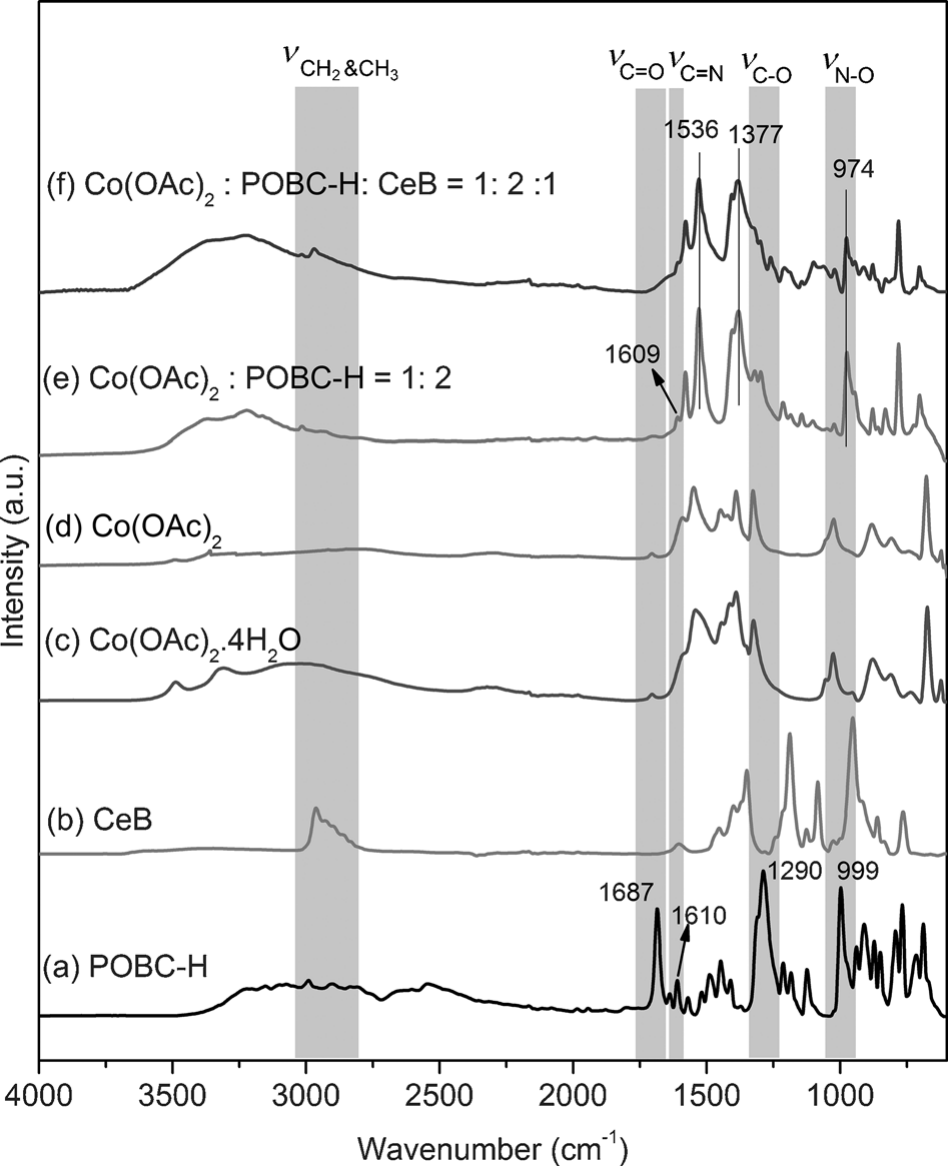
IR spectra of samples related to the Ce-POBC-Co single-source precursor.

Formation of the single-source precursor according to Equation (2) cannot be verified by means of NMR spectroscopy because of the paramagnetic properties of Co, and therefore only FTIR was used to monitor the reaction (see Figure 1).


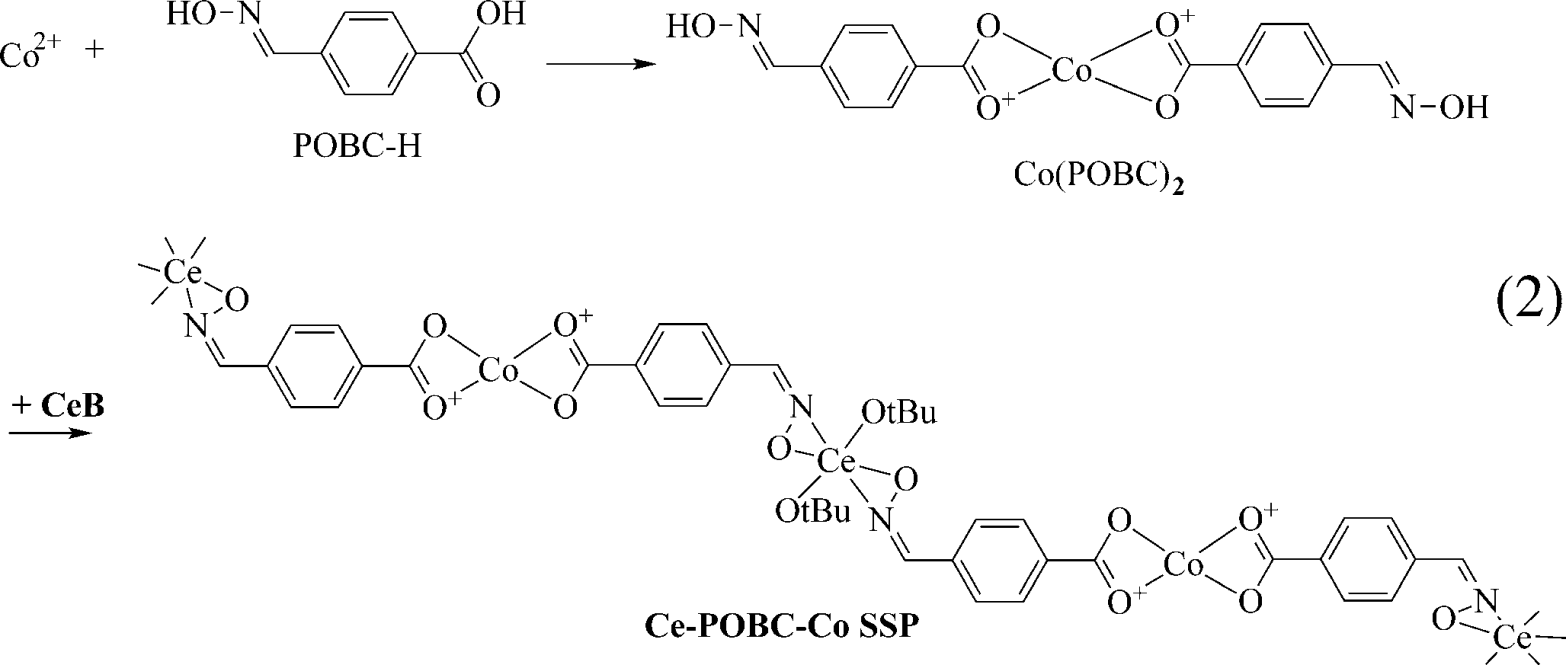
(2)

In the spectrum of POBC–H (Figure 1a), the bands at 1685, 1610, 1290, and 999 cm^−1^ correspond to *ν*_C=O_, *ν*_C=N_, *ν*_C–O_, and *ν*_N–O_, respectively. After reaction of POBC–H with Co(OAc)_2_ (0.5 equiv), the *ν*_C=O_ band was shifted from 1687 to 1536 cm^−1^ and that of *ν*_C–O_ from 1290 to 1377 cm^−1^ (Figure 1e). In contrast, the bands of *ν*_C=N_ and *ν*_N–O_ were almost unchanged, and the peak for the *ν*_C=N_ band was only shifted from 999 to 974 cm^−1^ for *ν*_N–O_. These changes in the IR spectra indicate that Co^2+^ was coordinated to the COO groups, whereas the NOH groups did not react at this stage [step 1 in Eq. (2)]. After addition of CeB to Co–POBC (Figure 1f), the intensities of *ν*_C=N_ and *ν*_N−O_ bands decreased, which suggested the reaction of the NOH groups with CeB and the formation of a Ce-POBC-Co single-source precursor [step 2 in Eq. (2)]. Whether one or two OR groups of CeB were substituted by oximate groups cannot be determined by IR spectroscopy. On the basis of previous investigations of oximate-substituted zirconium alkoxides, Zr(OR)_4−*x*_(O–N=CRR′)_*x*_,[10] and given the general similarity between Zr and Ce alkoxides, disubstitution as shown in Equation (2) is more likely. The degree of substitution, however, would hardly influence the outcome of sol–gel reactions.

### Materials characterization

**Figure 2:**
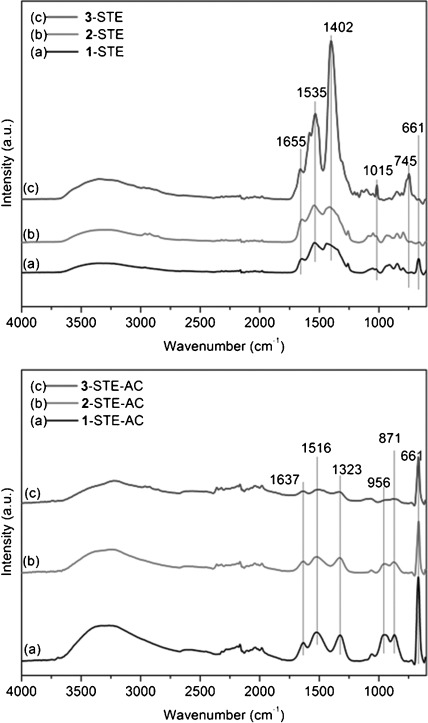
IR spectra of CoO_*x*_–CeO_2_ samples after STE treatment (top) and after STE-AC treatment (bottom).

The IR spectra of **1**-STE and **2**-STE were almost the same (Figure 2, top) except for the Co–O vibration at 661 cm^−1^ in sample **1**-STE, which indicates the formation of Co oxide species for **1**-STE already at this stage. The IR spectrum of **3**-STE is quite different, and the high band intensity of organic groups in the range of 1250–1750 cm^−1^ (COO: 1535, 1402 cm^−1^; N–O: 1015 cm^−1^) suggests that more organic groups were retained during solvothermal treatment.

**Figure 3:**
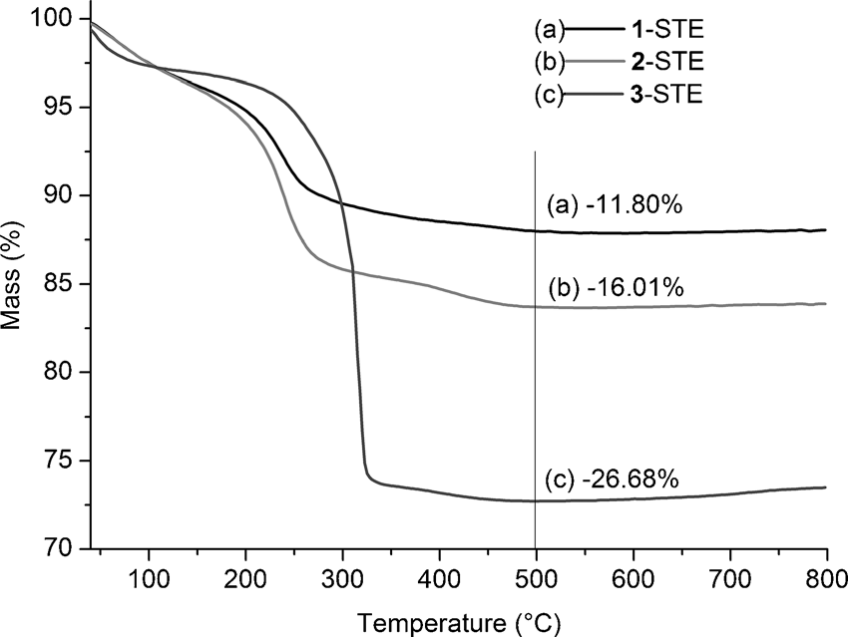
TGA of CoO_*x*_–CeO_2_ after solvothermal treatment.

This was also observed by means of thermogravimetric analysis (TGA; Figure 3), in which the weight loss of **3**-STE was 26.7 % owing to the presence of POBC or fragments thereof after solvothermal treatment. In each case, most of the organic components were removed below 350 °C, but there was still small weight loss up to 450 °C. The solvothermally treated samples (STE-AC) were therefore calcined at 500 °C for two hours to remove the organic constituents. In the IR spectra of the calcined samples (Figure 2, bottom), all samples have Co^2+^–O vibrations at 661 cm^−1^. The band intensity of sample **1**-STE-AC was the strongest, which indicated the formation of cobalt oxide species. Furthermore, the first overtones of Ce–O vibration (956 and 871 cm^−1^) were also observed for **1**-STE-AC and **2**-STE-AC, but not for **3**-STE-AC. Thus the band intensity change of the first Ce–O overtone might suggest the different dispersion of Co in sample **3**. The more homogeneously Co is dispersed, the lower the intensity of the first overtone is.

**Figure 4:**
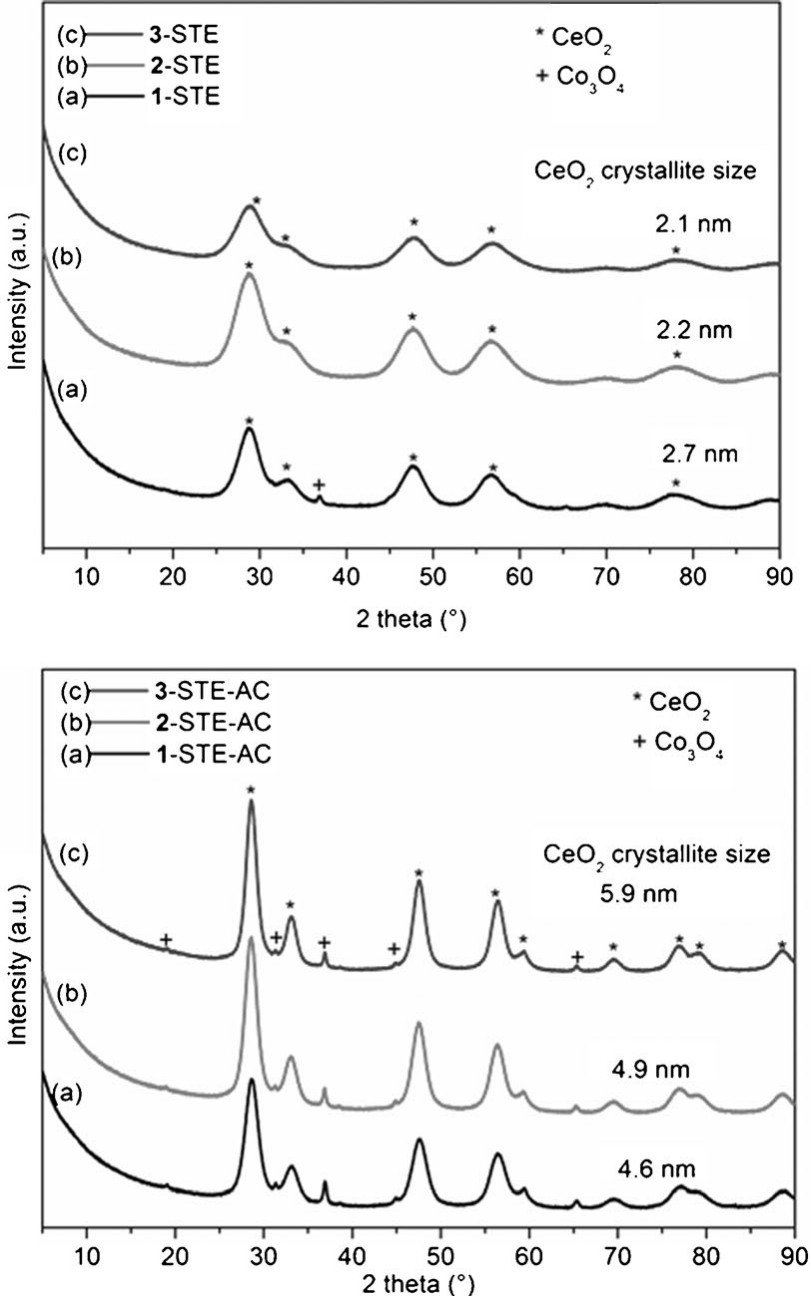
XRD patterns of Co_3_O_4_–CeO_2_, after STE treatment (top) and after STE-AC treatment (bottom).

Only **1**-STE showed weak reflections of Co_3_O_4_ in X-ray diffraction (XRD) after solvothermal treatment, in contrast to the other two STE samples with only CeO_2_ reflections (Figure 4, top). The diameter of the ceria crystallites, as calculated by Scherrer’s equation after baseline correction prior to the Gaussian fit, was in the range 2.1–2.7 nm. When the STE samples were treated in air at 500 °C for two hours, the crystallite size increased to 4.6–5.9 (see Figure 4). The smallest increase in size was for **1**. After calcination, all the samples showed weak Co_3_O_4_ reflections at about 37.5° (Figure 4, bottom). It was not possible to calculate the Co_3_O_4_ crystallite sizes because of the low intensity of the reflections.

**Figure 5:**
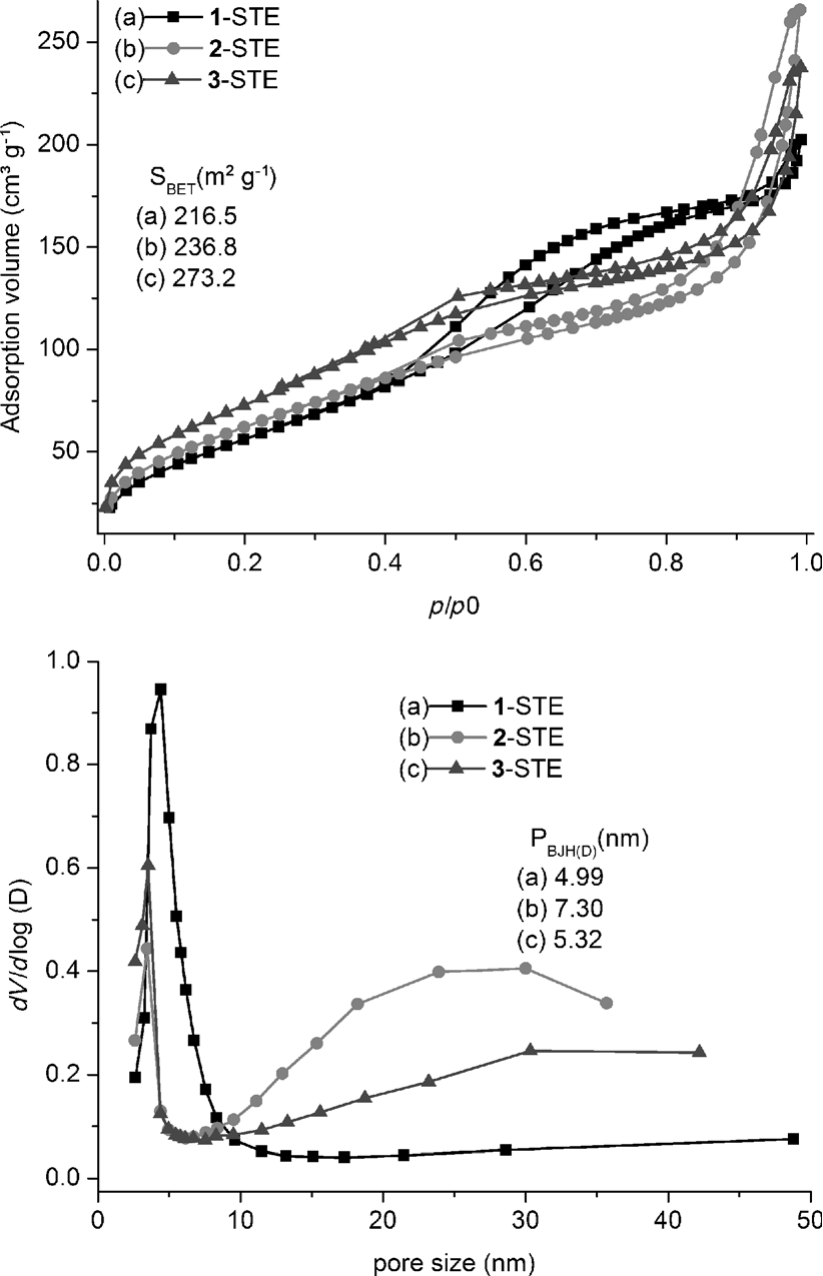
N_2_ adsorption–desorption isotherms (top) and pore-size distributions (bottom) after STE treatment.

According to N_2_ adsorption experiments (Figure 5), **1**-STE was mesoporous with an essentially monomodal pore-size distribution in the lower mesoporous range. In contrast, **2**-STE and **3**-STE had a bimodal pore-size distribution with pore sizes both in the lower and upper mesoporous range. All three STE samples had high surface areas in the range of 215–270 m^2^g^−1^ (see Figure 5).

**Figure 6:**
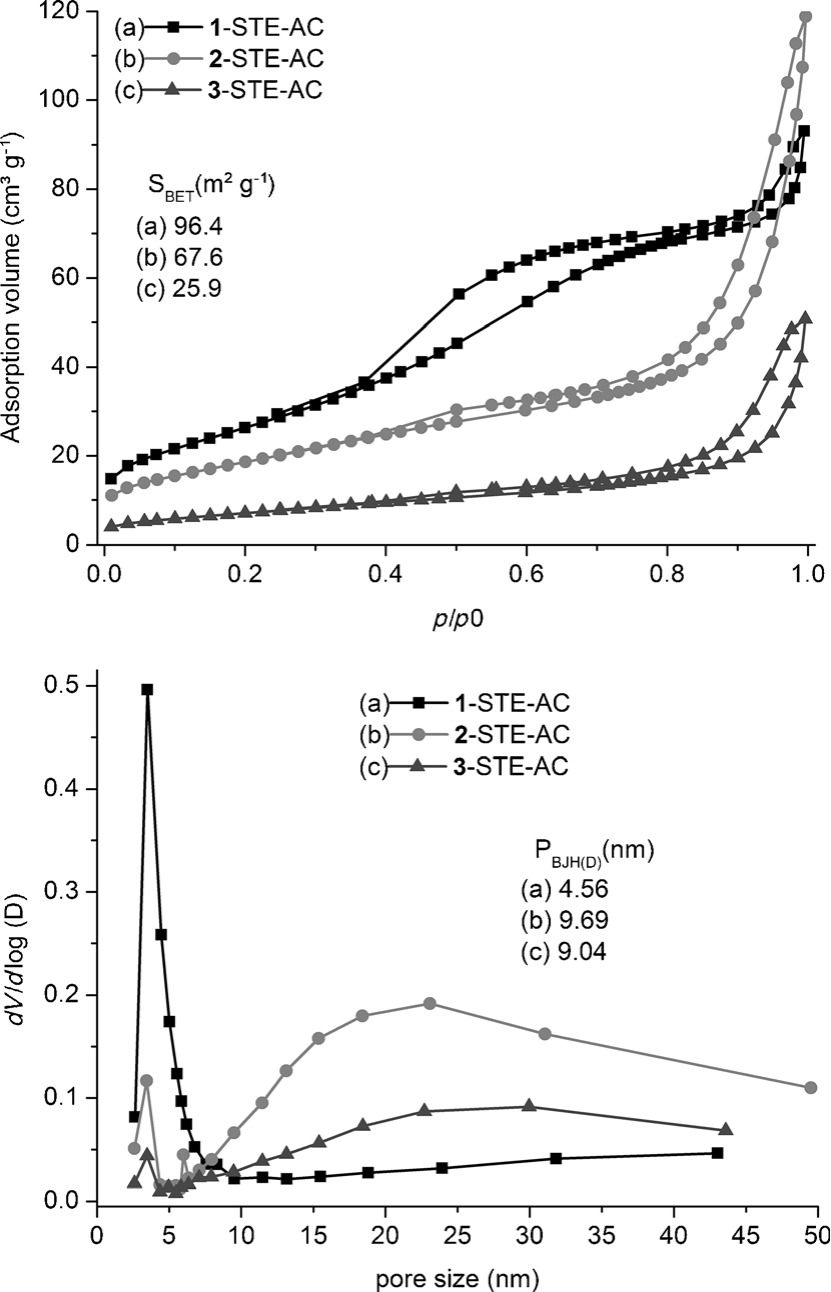
N_2_ adsorption–desorption isotherms (top) and pore-size distributions (bottom) for the STE-AC samples.

The pore-size distribution of **1**-STE-AC after calcination at 500 °C for two hours (Figure 6) was essentially the same. In **2**-STE-AC and **3**-STE-AC, however, the smaller pores had largely disappeared owing to the calcination process. In all samples the surface area decreased drastically to 25–96 m^2^g^−1^ upon calcination.

### Structural model

**Figure 7:**
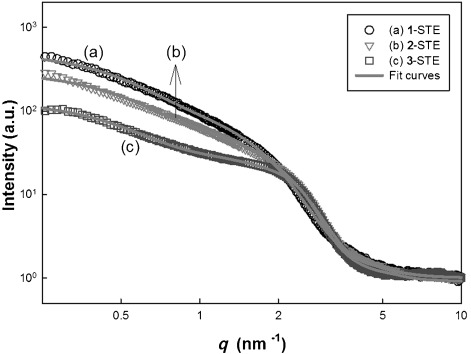
SAXS curves after STE treatment. The fits (gray lines) are fits for spheres with a bimodal size distribution with an additional small structure factor from a hard-sphere model for 3-STE to account for a weak agglomeration.

Since XRD did not provide information on the Co-containing structures before calcination, small-angle X-ray scattering (SAXS) measurements were carried out (Figure 7). A clear difference between the three samples is that **3**-STE has a more pronounced shoulder, which indicates homogeneous particles with a diameter of about 2.2 nm. Additionally, there is possibly some weak interaction between the particles in **3**-STE. To take this into consideration, we used an additional structure factor derived from a hard-sphere potential[11] for the fit curve. The weak interaction led to a low but not negligible hard-sphere volume fraction of 0.1. The unimodal distribution agrees with the literature, in which materials prepared from single-source precursors were reported to be more homogeneous.[2, 12] The intensity distribution for **1**-STE and **2**-STE is broader and has a considerably higher value towards low *q* values, which indicates a bimodal size distribution with a much higher amount of additional large particles. The SAXS results support that the nanoparticles are homogenous in the case of **3**-STE with a negligible amount of large particles, whereas pure CeO_2_ and CeO_2_ with CoO_*x*_ nanoparticles probably coexist for **1**-STE and **2**-STE. This leads to a broad additional distribution of nanoparticles with a larger size, the mean value being approximately twice the size of the pure CeO_2_ nanoparticles.

**Figure 8:**
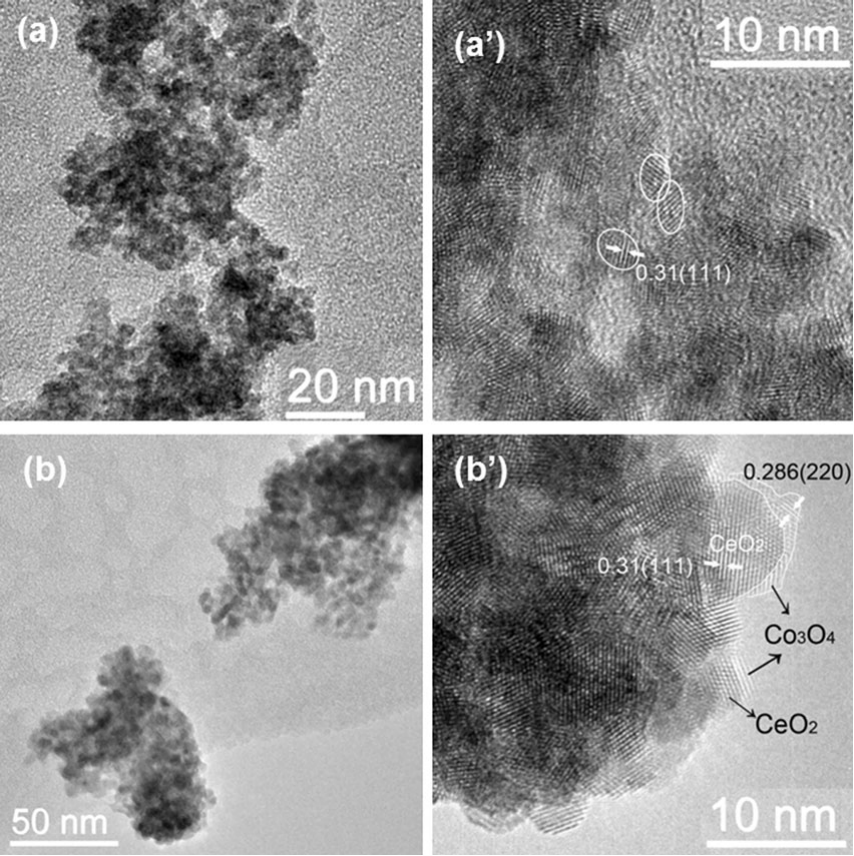
TEM and HRTEM images of a, a′) 1-STE and b, b′) 1-STE-AC.

**Figure 9:**
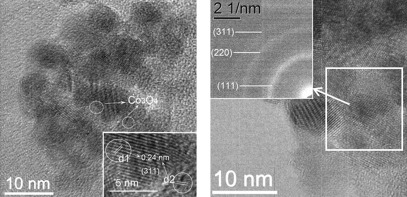
HRTEM images of 2-STE-AC (left) and 3-STE-AC (right). The inset in the right image is the selected-area electron diffraction (SAED) pattern.

All STE-AC samples were characterized by transmission electron microscopy (TEM) and high-resolution (HR) TEM (Figures 8 and 9). Because no CoO_*x*_ structures were detected in the STE samples by HRTEM (the XRD patterns showed that **2**-STE and **3**-STE contain no crystalline Co_3_O_4_, and **1**-STE contains only a very small proportion), only the image of **1**-STE is shown as an example (Figure 8).

The aggregates of **1**-STE and **1**-STE-AC (Figure 8a and b) are similar. The particle diameters in **1**-STE are about 3 nm with (111) lattice fringes (0.31 nm) that correspond to CeO_2_. No crystalline CoO_*x*_ phase was observed in HRTEM, although a weak Co_3_O_4_ diffraction peak was observed in the XRD pattern. This is probably due to the very small proportion of crystalline Co_3_O_4_. The size of most CeO_2_ particles of **1**-STE-AC is in the 4 to 7 nm range. At the perimeter of the particles, two different lattice spacings can be found (Figure 8a′). One lattice spacing is 0.31 nm, which corresponds to the (111) face of CeO_2_. The other lattice fringes with slightly brighter contrast have a distance of 0.286 nm, assigned to the (220) face of Co_3_O_4_.[13] Figure 8b′ shows that the Co_3_O_4_ crystallites are located on the surface of the aggregated CeO_2_ particles.

TEM images of **2**-STE-AC and **3**-STE-AC (not shown) are very similar to that of **1**-STE-AC (Figure 8). However, HRTEM showed that **2**-STE-AC (Figure 9, left) contains tiny Co_3_O_4_ particles (1–2 nm). Contrary to **1**-STE-AC, the Co_3_O_4_ particles are located in between the CeO_2_ particles. In contrast, no indications for crystalline Co_3_O_4_ (nano)particles were found for **3**-STE-AC (Figure 9, right), despite the very weak reflections of crystalline Co_3_O_4_ in powder XRD and the fact that the proportion of Co was the same as in the other two samples. Thus, because of the single-source precursor, the Co oxide particles are either amorphous or very small and well dispersed between the CeO_2_ grains.

**Figure 10:**
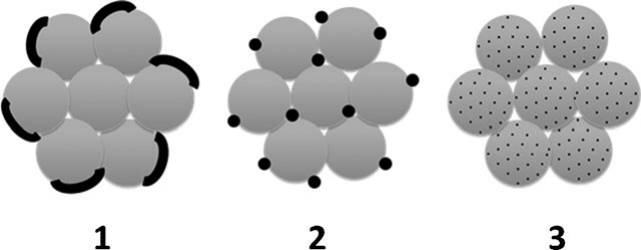
Suggested morphology of Co_3_O_4_–CeO_2_ after STE and AC treatment (gray: CeO_2_, black: Co_3_O_4_).

The HRTEM images clearly show that the CoO_*x*_ distribution in/on ceria strongly depends upon the preparation method. Co_3_O_4_ crystallites were formed both in **1**-STE-AC and **2**-STE-AC, but their distribution is different. In **1**-STE-AC, small Co_3_O_4_ crystallites were already present after STE treatment according to XRD (Figure 4). The HRTEM images indicate that the Co_3_O_4_ crystallites are preferentially located at the surface of the CeO_2_ particle aggregates, which apparently were already formed during sol–gel processing. For route 2, both Ce and Co precursors were present during formation of the sol, and CoO_*x*_ structures could be formed more easily in between the aggregated CeO_2_ grains. Moreover, up to 5 % Co can be incorporated into the CeO_2_ lattice.[14] Both HRTEM and SAXS investigations showed that the Co distribution in **3**-STE-AC is more homogeneous from the very beginning through all steps of the preparation process, as expected. The morphologies of Co_3_O_4_–CeO_2_ materials prepared by the three different methods are schematically shown in Figure 10.

### Catalytic properties

The catalytic activity of the solvothermally treated samples (STE) in CO oxidation reactions (5 % CO, 12.5 % O_2_, He balanced) is summarized in Table 1. We deliberately performed the catalytic measurements under conditions that are more relevant for technological applications, that is, by using as-received gases without drying. This means that H_2_O traces were present in the low parts per million level, which explains the activity onset above 100 °C (low-temperature activity can only be obtained using dry feed, which is technologically not very feasible). The *T*_10 %_ and *T*_90 %_ values, as well as *r*_130 °C_, *R*_130 °C_, and *R*_Co130 °C_, show that **1**-STE, which was prepared by the introduction of Co after formation of the ceria gel, is the most active and requires lower temperatures to oxidize CO completely than catalysts **2** and **3**, in which the Co was already introduced into the sol. The value of *R*_Co130 °C_ for **1**-STE is distinctly higher than that of 20 % Co_3_O_4_ on CeO_2_ prepared by conventional co-precipitation, in which *R*_Co130 °C_ is only around 1.2 mmol CO/mmol Co h^−1^.[15] Compound **1**-STE also has the lowest *E*_a_ (Table 1), which is similar to that of the noble metals supported on ceria, such as Pd/CeO_2_,[16, 17] Pt/CeO_2_,[16, 18] and Au/CeO_2_.[19] Thus, the combination of sol–gel and solvothermal methods allows one to obtain highly active CoO_*x*_-modified ceria catalysts. The low activity of **3**-STE is attributed to the high proportion of organic residues after solvothermal treatment, as evidenced by TGA (see Figure 3).

**Table 1:** Summary of CO conversion for Co_3_O_4_-modified CeO_2_

Sample	^[a]^*T*_10 %_ [°C]	^[b]^*T*_90 %_ [°C]	^[c]^*r*_130 °C_ [mol/s g^−1^]	^[d]^*R*_130 °C_ [mol/s m^2−1^]	^[e]^*R*_Co130 °C_ [mmol CO/ mmol Co h^−1^]	^[f]^*E*_a_ [kJ mol^−1^]
**1**-STE	117	155	2.48×10^−5^	1.14×10^−7^	68.7	47.4±0.3
**2**-STE	140	184	1.05×10^−5^	4.42×10^−8^	29.1	65.1±3.1
**3**-STE	194	244	8.37×10^−7^	3.06×10^−9^	2.3	77.5±4.3
**1**-STE-AC	131	163	1.48×10^−5^	1.53×10^−7^	41.0	61.8±1.8
**2**-STE-AC	133	173	1.39×10^−5^	2.06×10^−7^	38.5	58.0±1.7
**3**-STE-AC	142	186	9.25×10^−6^	3.57×10^−7^	25.6	65.9±2.3

[a] Reaction temperature for 10 % CO conversion. [b] Reaction temperature for 90 % CO conversion. [c] Reaction rate of CO oxidation at 130 °C per gram. [d] Normalized specific reaction rates of CO oxidation on a unit surface area at 130 °C. [e] Reaction rates based on the unit amount of Co at 130 °C. [f] Apparent activation energy.

Interestingly, the differences in the catalytic activity after calcination (STE-AC samples) were less pronounced (Table 1), although the order of activity was the same. This is clearly reflected in the *R*_130 °C_, *r*_130 °C_, and *R*_Co130 °C_ values. Taking the HTREM results (Figures 8 and 9) into account, a high proportion of Co oxide on the surface of (small) ceria particles apparently results in the highest catalytic activity. Compound **3**-STE-AC was still the least active composite for *R*_Co130 °C_, but the difference between it and **1**-STE-AC and **2**-STE-AC was distinctly smaller than before calcination. This is most likely due to the removal of the organic groups during calcination.

A more detailed investigation of the catalytic properties of **1**–**3** will be reported in the second part of this work.[6]

## Conclusion

Co_3_O_4_-modified CeO_2_ (Co/Ce 1:4) was prepared by combination of sol–gel and solvothermal methods. The way by which Co was introduced clearly influenced the morphology of the composites on the nanoscale. Using the single-source precursor Ce-POBC-Co resulted in a very homogeneous dispersion of Co oxide in the ceria matrix (samples **3**), as was expected from previous results for another system.[2] When a preformed ceria gel was impregnated with Co(OAc)_2_ under solvothermal conditions (samples **1**), Co_3_O_4_ particles were located on the surface of CeO_2_ particles or agglomerates thereof. Only in this case were weak XRD reflections of Co_3_O_4_ already observed after solvothermal treatment, in contrast to **2**-STE and **3**-STE. In the third variation, that is, sol–gel co-processing of CeB and Co(OAc)_2_, followed by solvothermal treatment, a highly dispersed mixture of CeO_2_ and Co_3_O_4_ particles was obtained.

Therefore the results clearly show that these variations of the synthetic route resulted in three different morphologies on the nanoscale, despite the same Co/Ce ratio, the same metal precursors, and the same processing steps (sol–gel processing and solvothermal treatment). The distribution of Co influenced the CeO_2_ crystallite particle size, the specific surface area, and the catalytic activity. Catalyst **1**-STE with small Co_3_O_4_ particles finely dispersed on the CeO_2_ surface exhibited the highest activity for CO oxidation, with an apparent activation energy of (47.4±0.3) kJ mol^−1^, which is comparable to ceria-supported noble-metal catalysts. A more detailed study of the catalytic properties—depending on the different morphologies—will be presented in the second part of this work.[6]

The outcome of the work can be generalized in the following way: Fine-tuning the synthetic parameters allows one to adjust a specific catalyst (composite) morphology on the nanoscale, with implications for the materials and catalytic properties.

## Experimental Section

### General

All solvents and solid chemicals were dried by standard methods. All operations that involved metal alkoxides were carried out under moisture-free and oxygen-free argon using standard Schlenk or glovebox techniques. Cerium *tert-*butoxide (CeB) was synthesized from (NH_4_)_2_[Ce(NO_3_)_6_] and sodium *tert-*butoxide in 1,2-dimethoxyethane (DME) according to literature procedures.[20, 21] The oily residue obtained after removal of the solvent was used directly for sol–gel processing without further purification. All solid chemicals were dried at <10^−3^ mbar for at least 5 h to remove H_2_O. Almost all H_2_O of Co(OAc)_2_**⋅**4 H_2_O was then removed, as shown in Figure 1. The dried cobalt acetate was dubbed Co(OAc)_2_ to differentiate it from Co(OAc)_2_**⋅**4 H_2_O. F127 and acetaldoxime were used as received.

### Synthesis of Ce-POBC-Co

*p*-Carboxybenzaldehyde oxime (POBC–H; 2 mmol)[22] and Co(OAc)_2_**⋅**4 H_2_O (1 mmol) were put into a Schlenk tube and dried at <10^−3^ mbar for 5 h to remove water. Then DME (5 mL) was added, and the mixture was stirred until a clear purple solution was formed, followed by the addition of a solution of CeB (1 mmol) in DME (5 mmol). During further stirring for 30 min the color of the solution changed to brown, which indicated the formation of the Ce-POBC-Co single-source precursor.

### Synthesis of Co3O4-modified CeO2

**Route 1**: AO (8 mmol) of was added to a solution of CeB (4 mmol) in DME (20 mL), and the mixture was stirred for 30 min. After the addition of F127 (0.025 mmol), stirring was continued for 1 h. No water was added during this stage. The sol was then deposited onto glass sheets (20×30 cm), which had been cleaned with 10 % NaOH, *i*PrOH, and acetone, then dried at 100 °C. The as-deposited films were exposed to ambient humidity at room temperature for 24 h. The obtained solid was then scraped off with a razor blade. The obtained gel powder (labeled **1**) was put into the Teflon liner of the autoclave (60 mL) and EtOH (30 mL) was added, followed by the addition of Co(OAc)_2_ (1 mmol) and stirring for 10 min. Then the autoclave was sealed and heated at 200 °C for 6 h. The product was washed at least three times with EtOH and H_2_O and finally dried at 105 °C overnight. The sample was labeled **1**-STE. Part of the sample was further heat-treated in air at 500 °C for 2 h (heat rate: 2 °C min^−1^) and was labeled **1**-STE-AC.

**Route 2**: AO (8 mmol) was added to a solution of CeB (4 mmol) in DME (20 mL) and the mixture was stirred for 30 min, followed by the addition of F127 (0.025 mmol) and stirring for 1 h. Then Co(OAc)_2_ (1 mmol) was added, and the mixture stirred for an additional 30 min. A gel powder (labeled **2**) was obtained as described for route 1, put into autoclave with EtOH (30 mL), and solvothermally treated at 200 °C for 6 h. The product was washed at least three times with EtOH and H_2_O and finally dried at 105 °C overnight. The sample was labeled **2**-STE. Part of the sample was further heat-treated in air at 500 °C for 2 h (heating rate: 2 °C min^−1^) and was labeled **2**-STE-AC.

**Route 3**: As the ratio of Ce/Co in the SSP is 1:1, an additional Ce source was needed to obtain a Ce/Co ratio of 4:1 in the final material. Firstly, SSP (1 mmol) was prepared as described above. In another Schlenk tube, AO (6 mmol) was added to a solution of CeB (3 mmol) in DME (15 mL) and the mixture was stirred for 30 min. Then the two solutions were combined, F127 (0.025 mmol) was added, and the resulting mixture was stirred for 30 min. The sol was treated similarly to that of route 2. The as-synthesized gel was labeled **3**, the solvothermally treated sample **3**-STE, and the calcined sample **3**-STE-AC.

### Characterization

TGA was performed using a Netzsch Iris TG 209C instrument with a platinum crucible and a heating rate of 10 °C min^−1^ under synthetic air. IR spectra were recorded using a Bruker Tensor 27 instrument with an ATR Micro Focusing MVP-QL instrument with a ZnSe crystal and using OPUS software version 4.0 for analysis.

Nitrogen-sorption measurements were performed using an ASAP 2020 instrument (Micromeritics). The samples were degassed under vacuum at 80 °C for at least 5 h prior to measurement. The total surface area was calculated according to Brunauer, Emmett, and Teller (BET) and the pore-size distribution (from the desorption branch) according to Barrett, Joyner, and Halenda (BJH).

XRD measurements were performed using a Philips X′Pert diffractometer with Cu_Kα_ radiation (*λ*=1.5406 Å).

High-resolution transmission electron microscopy HRTEM was performed using a TECNAI F20 instrument operated at 200 kV. The samples were ultrasonically dispersed in EtOH for 30 min before the measurements and then deposited onto copper grids covered with porous carbon films.

Small-angle X-ray scattering was performed with Cu_Kα_ radiation (*λ*=0.1542 nm) using a microfocus source (Incoatec IμS High Brilliance) and a 2D position sensitive detector (Vantec 2000, Bruker AXS). Before the measurements, the powder samples were dispersed in EtOH and ultrasonically deagglomerated for 30 min. Two positions of sample-to-detector distance allowed us to cover a range of the scattering vector *q* from 0.1 to 20 nm^−1^. All SAXS patterns were radially averaged to obtain the scattering intensities, which were dependent on the scattering vector *q*=4*π*/*λ*sin*θ*, in which 2*θ* is the scattering angle and *λ* the X-ray wavelength. A power-law background with slope *q*^−4^ (large objects>100 nm) was subtracted for each sample.

### Catalytic tests

CO oxidation reactions were performed using a continuous-flow fixed-bed quartz reactor under atmospheric pressure. The sample (10 mg), mixed with quartz powder to avoid mass- and heat-transfer limitations, was loaded into the reactor and pretreated with synthetic air (50 mL min^−1^) at 200 °C for STE samples or 400 °C for STE-AC for 30 min (heating rate 10 °C min^−1^). The sample was then cooled to 30 °C under a flow of synthetic air before 5 vol % CO, 12.5 vol % O_2_, and 82.5 vol % He mixture (total flow 50 mL min^−1^) were introduced. Then the system was heated to 250 °C with a ramping rate of 2.5 °C min^−1^. The concentrations of CO and CO_2_ in the outlet stream were monitored by gas chromatography using a HP-PLOT Q column and a flame-ionization detector.

## References

[1] Schubert U (2004). Adv. Eng. Mater.

[2] Yang J, Akbarzadeh J, Maurer C, Peterlik H, Schubert U (2012). J. Mater. Chem.

[3] Tang C-W, Wang C-B, Chien S-H (2009). Catal. Lett.

[b01] Woods MP, Gawade P, Tan B, Ozkan US (2010). Appl. Catal. B.

[b02] Liotta LF, Di CG, Pantaleo G, Deganello G (2007). Appl. Catal. B.

[b03] Dhakad M, Mitshuhashi T, Rayalu S, Doggali P, Bakardjiva S, Subrt J, Fino D, Haneda H, Labhsetwar N (2008). Catal. Today.

[4] Liotta LF, Ousmane M, Di CG, Pantaleo G, Deganello G, Marci G, Retailleau L, Giroir-Fendler A (2008). Appl. Catal. A.

[b04] Hou X-D, Wang Y-Z, Zhao Y-X (2008). Catal. Lett.

[b05] Liotta LF, Di CG, Pantaleo G, Venezia AM, Deganello G (2006). Appl. Catal. B.

[5] Tang C-W, Kuo M-C, Lin C-J, Wang C-B, Chien S-H (2008). Catal. Today.

[7] Yang J, Lukashuk L, Li H, Foettinger K, Rupprechter G, Schubert U (2014). Catal. Lett.

[b06] Yang J, Peterlik H, Lomoschitz M, Schubert U (2010). J. Non-Cryst. Solids.

[8] Lomoschitz M, Peterlik H, Zorn K, Baumann SO, Schubert U (2010). J. Mater. Chem.

[9] Artner C, Czakler M, Schubert U (2014). Chem. Eur. J.

[10] Baumann SO, Puchberger M, Schubert U (2011). Dalton Trans.

[11] Kinning DJ, Thomas EL (1984). Macromolecules.

[12] Torma V, Peterlik H, Bauer U, Rupp W, Hüsing N, Bernstorff S, Steinhart M, Goerigk G, Schubert U (2005). Chem. Mater.

[13] Yang L-X, Zhu Y-J, Li L, Zhang L, Tong H, Wang W-W, Cheng G-F, Zhu J-F (2006). Eur. J. Inorg. Chem.

[14] Wang J, Shen M, Wang J, Gao J, Ma J, Liu S (2011). Catal. Today.

[15] Kang M, Song MW, Lee CH (2003). Appl. Catal. A.

[16] Cargnello M, Doan-Nguyen VVT, Gordon TR, Diaz RE, Stach EA, Gorte RJ, Fornasiero P, Murray CB (2013). Science.

[17] Yee A, Morrison SJ, Idriss H (1999). J. Catal.

[18] Bera P, Gayen A, Hegde MS, Lalla NP, Spadaro L, Frusteri F, Arena F (2003). J. Phys. Chem. B.

[19] Shimada S, Takei T, Akita T, Takeda S, Haruta M (2010). Stud. Surf. Sci. Catal.

[20] Gradeff PS, Schreiber FG, Brooks KC, Sievers RE (1985). Inorg. Chem.

[21] Evans WJ, Deming TJ, Olofson JM, Ziller JW (1989). Inorg. Chem.

[22] Yang J, Puchberger M, Qian R, Maurer C, Schubert U (2012). Eur. J. Inorg. Chem.

